# A review on effective soil health bio-indicators for ecosystem restoration and sustainability

**DOI:** 10.3389/fmicb.2022.938481

**Published:** 2022-08-17

**Authors:** Debarati Bhaduri, Debjani Sihi, Arnab Bhowmik, Bibhash C. Verma, Sushmita Munda, Biswanath Dari

**Affiliations:** ^1^ICAR-National Rice Research Institute, Cuttack, India; ^2^Department of Environmental Sciences, Emory University, Atlanta, GA, United States; ^3^Department of Natural Resources and Environmental Design, North Carolina Agricultural and Technical State University, Greensboro, NC, United States; ^4^Central Rainfed Upland Rice Research Station (ICAR-NRRI), Hazaribagh, India; ^5^Agriculture and Natural Resources, Cooperative Extension at North Carolina Agricultural and Technical State University, Greensboro, NC, United States

**Keywords:** ecosystem restoration, ecosystem stability, resilience and resistance, soil health, bio-indicators, molecular bio-indicators

## Abstract

Preventing degradation, facilitating restoration, and maintaining soil health is fundamental for achieving ecosystem stability and resilience. A healthy soil ecosystem is supported by favorable components in the soil that promote biological productivity and provide ecosystem services. Bio-indicators of soil health are measurable properties that define the biotic components in soil and could potentially be used as a metric in determining soil functionality over a wide range of ecological conditions. However, it has been a challenge to determine effective bio-indicators of soil health due to its temporal and spatial resolutions at ecosystem levels. The objective of this review is to compile a set of effective bio-indicators for developing a better understanding of ecosystem restoration capabilities. It addresses a set of potential bio-indicators including microbial biomass, respiration, enzymatic activity, molecular gene markers, microbial metabolic substances, and microbial community analysis that have been responsive to a wide range of ecosystem functions in agricultural soils, mine deposited soil, heavy metal contaminated soil, desert soil, radioactive polluted soil, pesticide polluted soil, and wetland soils. The importance of ecosystem restoration in the United Nations Sustainable Development Goals was also discussed. This review identifies key management strategies that can help in ecosystem restoration and maintain ecosystem stability.

## Highlights

- A total of 250 reported studies (including original research, review papers, opinion papers, etc.) on ecosystem restoration and soil sustainability were reviewed.
- Forms of soil degradation were discussed along with important soil bio-indicators.
- A connection was established between soil health, soil sustainability, and ecosystem restoration.
- Management strategies were proposed to deal with altered soil scenarios.
- Advanced molecular techniques for assessing sensitive soil bio-indicators were briefed.

## Introduction

With the current advent of the changing climate, the state of environmental degradation and destruction of different ecosystems is considerably taking place on a “catastrophically short timescale” (Novacek and Cleland, 2001), which is estimated to accelerate from 1,000 to 10,000 times than the normal rate (Wilson, 1988). Biological diversity or biodiversity is of pivotal importance to sustain a better future. Humans have a great responsibility toward maintaining and enhancing global biodiversity at an ecosystem level. On a more anthropocentric level, natural ecosystems provide human society with food, fuel, and timber. Habitat loss is the leading cause of species extinction (Wilson, 1988) and ecosystem service decline (Daily, 1997). The two ways to reverse this trend of habitat loss are conservation of currently viable habitats and restoration of degraded ecosystems.

To understand the importance of ecosystem restoration, attention must be drawn to the cause of ecosystem degradation and its consequences. Ecosystem degradation is an outcome of long-term environmental degradation. By definition, environmental degradation is any change or disturbance to the environment perceived to be deleterious or undesirable. The deterioration of the environment is manifested in different ways through the depletion of resources, such as air, water, and soil resulting in habitat destruction, the extinction of wildlife, and pollution leading to the complete destruction of ecosystems. The degradation of the environment mainly occurs due to overexploitation of resources for short-term economic goals *viz*. deforestation for mining, building roads, or exploitation of flora and fauna. Some natural phenomena like active volcanoes, hurricanes, droughts, and earthquakes may also be responsible for causing damage to the ecosystem. Many of the world's ecosystems have undergone significant degradation with negative impacts on biological diversity. Fundamentally, ecosystem degradation is an environmental problem that diminishes the capacity of species to survive. Ecosystem restoration can be the only solution to the existing crisis.

Ecosystem restoration is essentially the “process of assisting the recovery of an ecosystem that has been degraded, damaged, or destroyed” (SER, 2004). The practice of ecosystem restoration includes a wide scope, such as erosion control, reforestation, usage of genetically local native species, removal of non-native species and weeds, revegetation of disturbed areas, daylighting streams, and reintroduction of native species, as well as habitat and range improvement for targeted species. The nutrient cycles and energy fluxes are the most basic and essential components of ecosystems (Ochoa-Hueso et al., 2021). An understanding of the full complexity and intricacies of these cycles is necessary to address any ecological processes that may be degraded. A functional ecosystem, which is completely self-perpetuating or natural, is the ultimate goal of restorative efforts (Farrell et al., 2021). Since these ecosystem functions are emergent properties of the system, monitoring and management are crucial for the long-term stability of an ecosystem.

The management of the ecosystem for its sustainability and stability calls for appropriate measures, such as monitoring of soil health particularly in degraded agricultural soils. It is already established that excessive use of inputs has degraded many fertile lands in the recent past. Therefore, it has now become more pertinent to identify and quantify the soil properties to arrest further degradation. To facilitate sound recommendations (for fertility restoration) to the farmers, robust information on soil health is required.

Over the years, ecosystem restoration has proliferated with several collaborative and multidisciplinary global projects called the following: 1. The EcoHealth Network (relies on the mutual benefits of ecological and human health), 2. The UN System of Environmental-Economic Accounting (SEEA EA); a standardized ecosystem accounting framework merging with economic frameworks, 3. The Natural Capital Project (NatCap), where global partnership existed between the wellbeing of people was aimed *via* ecosystem restoration and nature-based solutions, and 4. The INCASE project, to test the natural capital collaboration with scientists and stakeholders at a river catchment scale, was developed in Ireland (Farrell et al., 2021). Realizing the importance of the subject and growing interest in research, several conferences have been organized in the last few years; to name a few, “The 9th World Conference on Ecological Restoration” by the Society for Ecological Restoration (CIFOR, CGIAR, June 2021) and the National Conference on Ecosystem Restoration, by the University of Florida (July–August 2021) were also conducted recently.

Restoration ecologists and other conservation biologists agree that habitat is the most important locus of biodiversity protection. There has been a growing realization and appreciation in restoration ecology for the role of soil ecology in restoring and maintaining diverse biological communities both above- and belowground (Farrell et al., 2020; Cavender-Bares et al., 2021). Therefore, soil health restoration is important to consider while designing ecological restoration strategies. Soil dynamic properties can be used to monitor and assess the consequences of restoration activities on ecosystem functioning and services. In any case, finding suitable soil health indicators to monitor ecological restoration activities at different scales requires a full understanding of soil-plant–ecosystem relationships (Raiesi and Salek-Gilani, 2020). Current methods for assessing soil health fail to provide a complete picture of the status of the functioning soil system. Although knowing these aids management decisions, they lack any indication of the dynamic ways, in which soils need to respond to anthropogenic stresses and disturbances.

One of the ways to address this is to comprehensively determine soil microbial community characteristics and the biogeochemical properties they influence. To date, the exploration and application of soil ecological knowledge to restoration questions have been dominated by soil microbial ecology. Since micro-organisms could act directly or indirectly on organic matter decomposition and the promotion and maintenance of several soil properties, some characteristics of soil microbial communities have been used as ecological indicators of ecosystem disturbances and plant cover restoration (Gama-Rodrigues et al., 2008; Muñoz-Rojas et al., 2016); e.g., the microbial biomass is considered one of the most sensitive and effective indicators because it is directly influenced by biotic and abiotic factors (Karlen et al., 2019; Nunes et al., 2020). Soil microbial and biochemical properties, such as the metabolic quotient (qCO_2_) (ratio basal respiration: microbial carbon C) and microbial quotient (qMIC) (ratio microbial C: organic C), are also cited in the literature as efficient properties to evaluate the soil health (Maini et al., 2020; Simfukwe et al., 2021). On the other hand, soil biochemical properties are already established indicators of soil health, but there is still no consensus as to how they should be used. Generally, biochemical properties related to the biological cycling of the elements (carbon, nitrogen, phosphorus, and sulfur) are used to diagnose soil health. These properties include both general biochemical parameters (i.e., microbial biomass C, dehydrogenase activity, and nitrogen mineralization potential) and specific biochemical parameters (i.e., the activity of hydrolytic enzymes, such as phosphatase, urease, and β-glucosidase). Biochemical properties can be used both individually, as simple indices, or in combination using complex equations derived from mathematical combinations or the application of statistical programs (Lehmann et al., 2020). The results described in the literature for both are contradictory and question the validity of the use of biochemical properties as health indicators. Complex expressions, in which different properties are combined, are thought to be highly suitable for estimating soil health, although their use is limited to the area and situation, in which they have been described (Rinot et al., 2019; Thiele-Bruhn et al., 2020). Currently, there is a knowledge gap in the literature regarding the systematic compilation of the studies meant for the understanding of ecosystem restoration under various ecological conditions over the decades. The objective of this review article is to summarize some of the many attempts that have been made for the quantitative evaluation of soil health under various ecological constraints, including microbial and biogeochemical indicators, and proposed management strategies that improve ecosystem restoration measures.

So far, several studies have been conducted that specifically determine the role and impact of soil microbial and biochemical indicators under several land uses, management practices followed in the recent past or from several years, and stressed soil (drought and salinity, which change the soil's physical and fertility attributes) conditions in different parts of the world. However, the authors of this work felt the urge for a systematic and informative compilation of studies meant for the understanding of ecosystem restoration in various situations that may be suitable for global readers.

We have categorically emphasized the studies, which are solely based on identifying the bio-indicators, that were either influenced by the natural or the altered management systems. Future research priorities can be set for those who have focused on environmental management and fulfilling sustainable development goals. Our sole purpose behind this compilation is not only to gather literature but to also make our best attempt to analyze what progression was made over the years to identify the microbial and biochemical indicators under different situations that comes in *via* both thought process and technologically wise. We, the team of authors, have searched for 250 scientific kinds of literature (including original research, review papers, opinion papers, etc.) over three decades from different corners of the world in this aspect, and have compiled and presented it suitably to the global readers. In the present scenario, it is more important to assess where we are, and in which direction we are leading for ecosystem restoration.

This review summarizes the many attempts that have been made at the quantitative evaluation of soil quality (based on the selected soil indicators) that eventually relate to ecosystem restoration of agricultural soil and other degraded soils and how important the role of microbial and biochemical indicators is in the whole process. Measuring the soil quality, restoration, and resilience are all realized as interconnected and point toward a sustainable soil system, concerning both agriculture and the environment. Over and above, a brief discussion on proposed management strategies and how they can uplift the ecosystem restoration status also took place in our compilation. The prominence of ecosystem restoration in Sustainable Development Goals as proposed by the United Nations was also highlighted.

## Concept of microbial/biochemical indicators

There are many definitions of “Bio-indicators” if we search in the literature. Moreover, it has been slightly modified from time to time. Hodkinson and Jackson (2005) mentioned that “a bio-indicator is a species or a group of species that reflects biotic and/or abiotic levels of contamination of an environment,” whereas Stankovic and Stankovic (2013) described, “a bio-indicator is an organism or a part of an organism or a community of organisms, which contains information on the quantitative aspects of the quality of the environment; exposure of organisms can be measured by either levels or effects.” So far, it is established that soil microbial parameters, which provide information on the biomass, activity or functionality, and diversity of microbial communities have been widely proposed as bio-indicators of soil health (Gómez-Sagasti et al., 2012). Bio-indicators play a substantial role, together with common soil chemical indicators, in the reclamation of soil health. However, not much attention has been paid to identifying the set of ideal/sensitive bio-indicators for stressed or problematic soils, which demands equal attention for soil remediation or restoration, a globally important sustainability issue.

**Figure 1:**
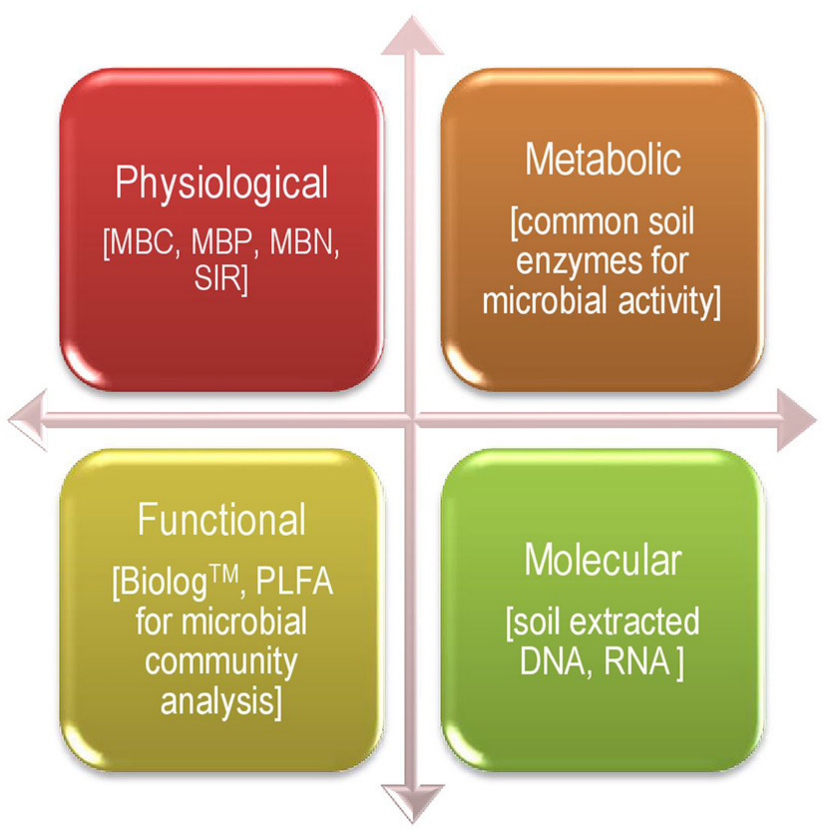
Soil bio-indicators as classified on the basis of techniques of measurement.

These days, an extensive number of techniques/approaches are frequently involved to estimate common soil microbial parameters (bio-indicators), and, hence, they can be broadly classified into four categories: 1. Physiological, 2. Metabolic, 3. Functional, and 4. Molecular (Figure 1). There are good numbers of bio-indicators that were investigated for agricultural systems and used for indexing soil health and sustainability under long-term agro-ecosystem (Masto et al., 2007; Bhaduri and Purakayastha, 2014; Bhowmik et al., 2016, 2017a; Bhaduri et al., 2017a; Fortuna et al., 2018a,b) at altered management practices in a wide range of agricultural soils (Koper and Piotrowska, 2003; Kang et al., 2005; Bhattacharjya et al., 2017), such as a climate-dominated natural soil (Bastida et al., 2006), pesticide-afflicted soils (Saha et al., 2015, 2016a,b), hydrocarbon-polluted soil, and a mine–reclaimed soil (Mukhopadhyay et al., 2016). All possible relations between soil health and plant-microbe interactions in the plant rhizosphere with a special mention of soil bio-indicators have been explicitly discussed in other literature too (Bhaduri et al., 2015, 2017b, 2018; Bhowmik et al., 2017b, 2019).

## Methods

The idea of the review was conceived by the lead author who felt the need for such systematic compilation, and, subsequently, the team of authors was formed. After rounds of group meetings, the layout of topics was finalized, and the authors started to search for relevant information on different time scales. For searching relevant literature Google Scholar was trusted as a search engine. The authors used some common keywords, such as soil bio-indicators, ecosystem restoration, polluted soils, problem soils, land use intensification, agricultural sustainability, microbial community, soil and ecosystem responses, soil nutrient cycling, wetland pollution, and sustainable development goals. Systematic classification of published literature was grouped as per the major headings of the review. After the finalization of the first draft, the topics were redistributed for all possible checks among authors, and further refinement was done wherever possible. Two authors in the team dedicated their time to editing and the manuscript was finalized thereafter. At the time of the revision, a team of authors addressed all comments by anonymous reviewers.

## Results and Discussion

### The behavior of bio-indicators under distinct soil scenarios

Soil microbial assemblages and functions are robust indicators of environmental responses to disturbances in managed and natural ecosystems. However, the choice of keystone soil microbial indicator(s) can be calibrated with the type of scientific questions to be addressed (Siddig et al., 2016; Schloter et al., 2018). In this review article, we report potential soil microbial indicators that can serve as early warnings of ecological responses to a range of conditions, including land-use intensification and land management practices, pollution of heavy metal and organic wastes, ecosystem restoration projects, and environmental disturbances.

### Land use intensifications and land management practices

Traditional microbial community analysis, along with soil biochemical and biogeochemical analyses, can be fruitful in investigating the soil and environmental consequences of long-term farming practices (García-Orenes et al., 2016). Kumar et al. (2018) recently suggested the use of 16S rRNA amplicon sequencing-based operational taxonomic units (OTUs) to demonstrate that the community structure of beneficial soil bacteria like diazotrophs, which are known to increase in fields, receive balanced fertilization as compared to the fields receiving single nutrient, such as nitrogen. Changes in soil microbial community composition and structure can be indicative of land-use intensification, such as the conversion of native rangeland to silvopasture or sown pasture and subsequent alteration of plant cover and nutrient inputs (Xu et al., 2017). The occurrence of keystone taxa, however, can also inform about the influence of land management practices on the structure of soil (and root) microbial network, thus, soil and ecosystem functions (Bhowmik et al., 2016; Banerjee et al., 2018, 2019).

The sensitivity of microbial structural diversity as a function of land-use intensification can vary based on the ecosystem (or, dominant vegetation) type. For instance, intensive soil management practices in an agricultural system in Brazil greatly reduced the value of indices for soil microbial diversity (Siqueira et al., 2014), but those indices were not effective to explain the ecological consequences of land use intensifications in tropical rainforests of Amazonia (de Carvalho et al., 2016). The functional potential of the soil biota represented by the active fraction of the microbial community in the Biolog assay can serve as an alternative indicator to assess the influence of land use intensifications (Gomez et al., 2004). Other than vegetation types, changes in soil microbial diversity as a function of land-use intensification can also be contingent on biome types or climate and underlying edaphic properties (Houlbrooke et al., 2011; Trivedi et al., 2016).

A recent study indicated that agricultural intensifications could alter the expressions of soil biochemical indicators (e.g., enzyme activity) quicker than soil microbial diversity indicators (Pérez-Brandán et al., 2016). To that end, soil enzyme activities are often used as effective indicators for assessing the environmental consequences of applications of agricultural chemicals for soil nutrition (Gaind and Singh, 2016; Sihi et al., 2017) and xenobiotic pesticides (Sahoo et al., 2016; Mahapatra et al., 2017). One can also evaluate the ecological consequences (e.g., soil carbon loss) of land use management intensities, along with a wide spatial gradient of edaphic factors, using microbial ecophysiological traits, including growth efficiency, qCO_2_, and indicator proteins (Malik et al., 2018). Hereby, we discuss the use of specific bio-indicators for a wide range of soil ecosystems:

#### Mine deposited soil and its reclamation

Mine-deposited or mine-reclaimed soil has a close association with heavy metal toxicity. Hence, only a few kinds of literature cited some work in a similar direction. Earthworm (*Eisenia foetida*), as a bio-indicator, has been tested to assess Hg-toxicity and bio-availability in mine tailings. In another study, acidophilic bacteria were identified after 16S-rRNA profiling of bacterial community from extremely and moderately acidic lead-zinc mine tailing samples. A study from an Indian mine located at Ranigunj coalfields, dealt with environmental soil quality index, screened the bio-indicators, and differentiated their behaviors at underground mine (better dehydrogenase and fluorescein activities) and open cast mine soils (peroxidase activity) (Masto et al., 2015).

Rehabilitated soils include soils under cultivation or any productive use of mankind after keeping barren/fallow for a few to several years. Hence, their structure and properties are altogether different as compared to other soils. An early study (Majer, 1983) discussed that ants can serve as good bio-indicators owing to numerical abundance, size, and species richness, especially in the case of mine-rehabilitated soils. Another study also supported this finding showing that a close association exists between aboveground ant activity and belowground decomposition processes in terms of soil microbial biomass carbon at mine-disturbed sites in Northern Territory, Australia. This provided evidence that ants could be used as indicators of restoration success, following disturbance (Andersen and Sparling, 1997). In other instances, for long-term heavy metal polluted (Zn, Pb, Cd toxic) soil, urease and invertase enzyme activities were identified as “progress indicators” for soil rehabilitation (Ciarkowska et al., 2014). Schimann et al. (2012) proposed the ratio of soil Denitrifying Enzyme Activity (DEA) and Substrate Induced Respiration (SIR) as ecosystem indicators after disturbances of gold mining in tropical rainforests of French Guiana.

Fatty acid methyl ester (FAME) biomarkers can be used effectively to evaluate the reclamation progress of surface mines. Within this context, Mummey et al. (2002) observed an increased ratio of bacterial: fungal FAME biomarkers that also co-occurred with an increment of SOM content, indicating improved soil health due to ecosystem recovery after surface-mine reclamation. Interestingly, Anderson et al. (2008) suggested using MBC instead of soil organic carbon (SOC) as an indicator of ecosystem stability from surface coal mining in a semiarid area, even after a decade-long reclamation management practice. However, MBC alone may not always be a sensitive indicator of soil restoration in heterogeneous mountain systems like Pa'ramo in the High Tropical Andes (Abreu et al., 2009). Hence, bio-indicators should be carefully chosen for restoration project monitoring based on the prior knowledge of their degree of similarities and dissimilarities between reclaimed and pristine sites of similar geographic territories (White and Walker, 1997).

#### Trace (heavy) and organic pollutant affected soils

Expressions of stress proteins (AKA heat shock proteins), especially hsp70 and hsp60, have long been used for biomonitoring of exposure of soils to trace (or, heavy) metals and organic pollutants (Huggett, 2018). Ghotbi and Morgan (2007) synthesized the applicability of stress proteins expressed by soil bacteria under stressed conditions, such as sudden fluctuations in soil pH after metal contamination. Heavy metal contaminations are also known to increase the abundance of low molecular weight proteins in soils (Zhang et al., 2013). Stimulation of metal-resistant microbes is another widely observed response to heavy metal contamination in soil (Doleman, 1994; Shi et al., 2002). The effects of heavy metals on soil microbial communities can also be found to be reported in studies conducted by Zhao et al. (2014) and Chu (2018).

The abundance of functional genes related to fatty acid metabolism (e.g., *acc, fab*, and *fad* genes) can be used to evaluate contamination of organic biomolecules like hydrocarbon, especially in soils subjected to oil contamination from industrial effluents (El-Bestawy et al., 2005). Taxonomic profiling of soil microbiome informed by 16S rDNA analysis can also be used to evaluate the hydrocarbon exposure to soil, where the enrichment of Proteobacteria, Gamma Proteobacteria, and Bacteroidetes may indicate potential effects of the oil spill. A more traditional soil microbial diversity indicator like the Shannon diversity index also served as a useful indicator of soil contamination in a recent study conducted by Patel et al. (2016). Additionally, Galitskaya et al. (2015) indicated that metabolic quotient and cellulase activity are two very sensitive bio-indicators of radioactive oil waste.

There are several bio-indicators reported from heavy metal polluted soils. Various organisms, including microbes, fungi, plants, animals, and humans, are being used to identify and monitor the level of toxic metals in the ecosystem, encompassing air, water, sediment, soil, and the food chain Stankovic et al. (2014). Nematode diversity is a frequently used parameter, where maturity index (MI) is an established bio-indicator to assess the degree of heavy metal polluted soil (Coleman et al., 1998; Korthals et al., 1998). A recent report by Martin et al. (2018) mentioned *Pinus halepensis* trees as bio-indicators of heavy metal pollution, where tree rings and barks were used to evaluate environmental contamination. Another study reported that Cryptogamic biota, e.g., lichens and bryophytes, are useful to identify the degree of heavy metal toxicity in Zn-Pb polluted sites (Rola and Osyczka, 2018).

#### Desert soil/barren lands

Few researchers also attempted to identify promising bio-indicators for desert soils or barren lands. They have opined that rain frequency and continuity were determinants for desert soil under a xeric moisture regime that may cause changes in soil conditions and, the substrate utilization pattern of the microbial community as determined by the MicroResp^TM^ analysis (Saul-Tcherkas and Steinberger, 2009). Guan et al. (2015) concluded that soil nematode communities serve as bio-indicators under sandy ecosystems, whereas the plantation of *Caragana microphylla* improved nematode diversity. Another study stated that there was a beneficial impact of sand-stabilizing shrubs for various hydrolase and oxidase enzymes involving soil C-cycle (polyphenol oxidase, cellulose, and β-glucosidase) and N-cycle (nitrate reductase and urease) conducted at Tengger Desert, China (Hu et al., 2016).

#### Anthropogenic stressed soils

Soil invertebrate species are often reported as useful bio-indicator for monitoring qualitative and quantitative environmental changes in soil due to anthropogenic activities (Paoletti et al., 1996). Pollution creates a shift in the community structure of nematodes; for light to moderate pollution, the abundance of sensitive species is reduced while keeping the abundance of tolerant species unaffected; whereas the decrease in tolerant species is noticed under heavily polluted soils, due to toxic effects or a decline in microbial activity (Korthals et al., 1998).

#### Radioactive materials polluted soils

Radioactivity leaves a long-term footprint on soil ecological behavior. Although not many, few researchers revealed some interesting facts and findings. Zaitsev et al. (2014) reported after extensive radioecological research around the historic Chernobyl site (in and around Russia, Ukraine, Belarus, and Kazakhstan) that earthworms, millipedes, collembolans, and oribatid mites were the most appropriate bio-indicators of different radioactivity levels and types of radioactive pollution. A recent report indicated that radioactivity could induce biological effects in soil earthworms (family: Lumbricidae) *viz*. impairment of reproduction of individuals and reduction in population density; however, a higher survival rate of *Aporrectodea caliginosa* was observed after additional acute γ-irradiation with a dose of 2,270 Gy, which may be a sign of adaptation for higher doses of ionizing radiation and establish them as bio-indicator under soil contamination with radionuclides and heavy metals (Rybak et al., 2021). Another study reported that soil macro-invertebrates (e.g., ground beetle) served as important bio-indicators with 3–37 times lower abundance, and biodiversity at the contaminated sites enriched with uranium and arsenic over the control soil (Gongalsky, 2003). Gaso et al. (1995) discussed the role of land snails (*Helix aspersa* Müller) as bio-indicators in sites contaminated with radionuclides (^226^Ra, ^137^Cs, and ^40^K) for two decades. Aquatic mosses were also identified as bio-indicators of radioactive contamination (Hongve et al., 2002).

#### Pesticide polluted soils

Pesticides help control agricultural pests but often affect non-target soil biota and their processes (Pimentel, 1995; Aktar et al., 2009). The fate of the pesticide in the soil system depends on the chemical composition of the pesticide along with the soil's biotic and abiotic conditions (Pal et al., 2006). Depending on the transformation, the products could interact and disrupt the biological processes in the soil, thereby, influencing bio-indicators of soil health. However, most of the studies have found discordant results because of numerous interacting factors and complex soil dynamics. Researchers have investigated the effect of different types of pesticides, including insecticides, fungicides, and herbicides, and found that they have a positive or negative effect on soil microbial biomass (Chowdhury et al., 2008; Saha et al., 2015; Kumar et al., 2020). Repeated application of pesticides has been reported to significantly lower the soil microbial biomass, mainly the fungal populations, and has been reported to increase certain bacterial populations (Smith et al., 2000; Pal et al., 2006; Singh et al., 2015). Molecular techniques like DGGE were also to determine the effect of specific pesticides on the soil microbial community (Lo, 2010). They found that carbofuran stimulated the population of *Azospirillum* and anaerobic nitrogen fixers in flooded and non-flooded soil. However, specifically in non-flooded soils, butachlor reduced the population of *Azospirillum* and anaerobic nitrogen fixers. Soil respiration can serve as a bio-indicator to evaluate the effect of these chemicals on microbial CO_2_ respiration. Many studies reported the favorable effect of pesticide application on microbial growth and activity, whereas few studies show the adverse effect of pesticides on microbial respiration (Jail et al., 2015). Microbial metabolic quotient was found to increase due to chemical pesticide application indicating the requirement for microbes to use more energy for maintenance. The literature is replete with the effect of pesticide application on soil enzymatic activity (Saha et al., 2016a,b; Sahoo et al., 2017). Enzymes involved in nutrient cycling, including dehydrogenase, were mostly inhibited, whereas cellulose activity was stimulated due to pesticide application (Tu, 1992; Haney and Senseman, 2000; Chowdhury et al., 2008; Riah et al., 2014). Most of the studies to date have focused on soil incubation experiments but not much on field studies.

#### Wetland assessment and restoration

Like many other natural and managed ecosystems, the use of microbial metabolic indicators, coupled with hydric soil indicators, has been suggested for designing assessment strategies in wetland ecosystems (Merkley et al., 2004). Mieczan and Tarkowska-Kukuryk (2017) recently demonstrated the efficacy of using microbial loop components (or, microbial food webs) as a monitoring tool for evaluating ecological disturbance in a restored carbonate-rich fen. On the other hand, Dziock et al. (2006) reported the usefulness of using microbial diversity and community comparison indices to evaluate the effect of wetland pollution. Ratios of oligotrophic:copiotrophic organisms, such as the ratio of ammonia-oxidizing archaea (AOA) to ammonia-oxidizing bacteria (AOB), can be used to assess the success of wetland restoration projects (Martens-Habbena et al., 2009). To that end, Sims et al. (2012) recently proposed the use of the ratio of ammonia monooxygenase (*amoA*) gene copies for AOA to AOB for evaluating the effect of the nutrient loading in oligotrophic wetlands, where an increased value of AOA: AOB gene copies may indicate healthy conditions in those wetlands.

Molecular technologies like nucleic acid fingerprinting, especially terminal restriction length fragment polymorphism (tRFLP) and fluorescence *in situ* hybridization (FISH), along with microbial community-level physiological profile (CLPP), are also generally used as indicators for wetland trophic status (Castro et al., 2002; Sizova et al., 2003; Costa et al., 2007). Lipid biomarkers based on phospholipid fatty acid (PLFA) analysis also hold promise for assessing the efficacy of wetland restoration and management projects (Sims et al., 2013). A greater abundance of fungal biomarkers and increased ratios of gram-negative: Gram-positive bacteria served as an indicator of the restoration status of a calcareous subtropical wetland of Florida Everglades (Inglett et al., 2011). In contrast, a higher prevalence of spore-forming and stress-tolerant gram-positive bacteria like *Actinomycetes* can indicate the onset of environmental stresses, including extreme oligotrophy, drought, or warming in wetlands (Saetre and Baath, 2000; Yao et al., 2000).

### Impact of bio-indicators on soil nutrient cycling, soil quality, and ecosystem viability *vis-a-vis* sustainability

Soil is one of the most important natural resources, which determines the existence of plants and animals and governs human civilization. Soil conservation and maintenance are prerequisites for the environment and ecosystem stability. Soil degradation is a common phenomenon and is caused by different natural and manmade factors; hence, its restoration needs to be assured naturally or by human intervention. However, if we do not care for it properly, then soil degradation would reach an advanced stage, and its restoration would become practically difficult as there will be a need for site-specific techniques (conservation agriculture and integrated nutrient management) of restoring soil quality with a strategy to increase soil, water, and nutrient use efficiency (Lal, 2015). Quantification of soil degradation *vis-à-vis* restoration needs some measurable properties that can define the extent of the process. Nowadays, soil as a medium of plant growth is mainly defined in terms of its quality or health.

Soil is a heterogeneous, porous, living, and natural and dynamic system, which is crucial to maintaining the entire ecosystem, and its importance in crop production is judged by its inherent capacity to support crop growth and is reflected by soil quality and health, which is governed by different soil properties. There is always a debate in using the terms soil quality and soil health being defined differentially by different workers in several ways. The broad definition of soil quality as proposed by the Soil Science Society of America (SSSA) is “The ability of a specific type of soil to function within natural or managed ecosystem boundaries, to sustain plant and animal productivity, and maintain or improve air quality and water to support human health and livable” (Karlen et al., 1997). In other ways, soil quality can also be defined as the “fitness for use,” “capacity of a soil to function,” “ability to sustain productivity and maintain environmental quality,” etc. (Lal, 1993; Acton and Gregorich, 1995; Karlen et al., 1997). The quality of the soil depends on the climatic conditions of the region, soil characteristics, vegetation, anthropogenic influence, management strategy, etc., and complex interaction among them. Soil management and cropping practices are commonly known to alter the factors affecting almost all the biological processes in the soil, as well as soil quality (Bhaduri and Purakayastha, 2014). As a complex functional state, soil quality cannot be measured directly, but it may be inferred from management-induced changes in soil properties, better known as soil quality indicators (the measurable or quantifiable soil attributes). However, these indicators are neither well-defined, nor the set of accepted or approved parameters to characterize or define soil quality exist. The choice of soil attributes and its interpretation of measurements are not simple and straightforward because of their complexity and site-specificity (Bünemann et al., 2018). Moreover, it would be unrealistic or impossible to use all soil attributes as indicators, so a minimum set of soil attributes (better known as the minimum data set) encompassing chemical, physical, and biological soil properties are selected for soil quality assessment (Larson et al., 1994).

#### Biological indicators for soil nutrient cycling and soil quality

Apart from physical and chemical properties, soil quality, or soil health is also very closely linked to its biological properties, which consist of different attributes like microbial diversity, microbial biomass, enzyme quantities and activities, mineralizable carbon, nitrogen, phosphorus and sulfur, soil respiration, soil organic matter/carbon, and labile carbon pools, etc. It has been well-established that biological properties respond more rapidly to changes in agricultural management practices or environmental changes (Doran et al., 1996; Bhowmik et al., 2019; Kumar et al., 2019). In this context, biological indicators might be important and sensitive indicators to evaluate the effect of different management practices on soil health. The parameters commonly used for soil health assessment are mainly focused on soil properties, which change significantly with time after changes in management practices. It is well-understood that some of the soil's physical, as well as chemical, parameters will take significantly more time to show their changes as compared to biological parameters. So, soil biological parameters could be treated as early indicators for soil health changes. Soil organisms and microbial activity are assumed to be directly responsible for soil ecosystem processes, especially in the decomposition of soil organic matter and related nutrient cycling and transformations, which is further helpful to meet the plant mineral nutrition (Jacoby et al., 2017). All these components are regarded as major components in the global cycling of materials, energy, and nutrients.

**Figure 2:**
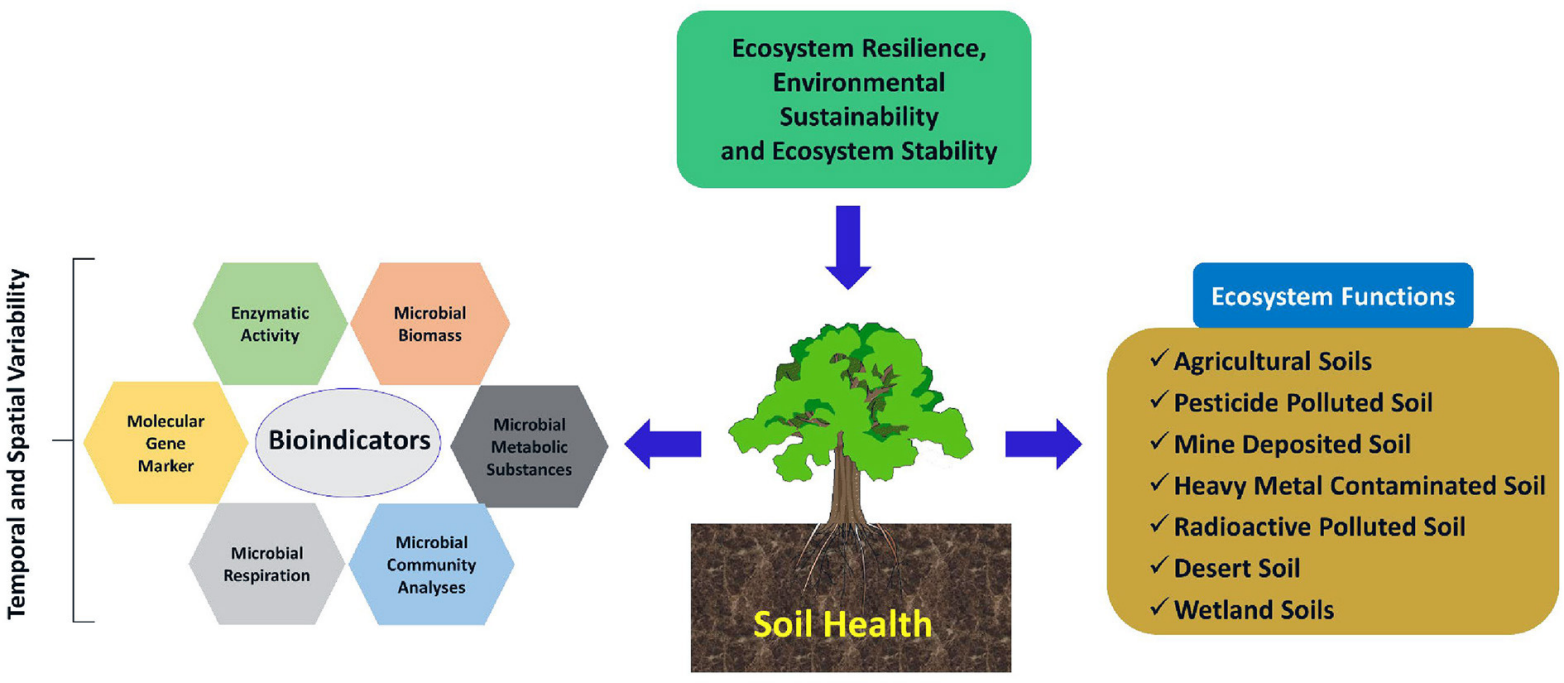
Schematic representation of soil health as an indicator of ecosystem resilience and stability. Soil health can be quantified and qualified in terms of various bio-indicators (e.g., Microbial biomass, respiration, metabolic substances, and community analyses as well as some enzymatic activity and molecular techniques) both at temporal and spatial scales. Multiple soil management system can strategize as a critical factor to determine the response mechanism of different bio-indicators.

Among the different soil biological properties, Bhaduri et al. (2017a) observed that soil respiratory quotient was a key indicator of soil biological processes, indicating the carbon balance in soil across contrasting input management in the rice-wheat cropping system. However, dehydrogenase activity occupied the second position to influence soil biological quality, as revealed from the principal component analysis. Therefore, there is growing evidence that soil microbiological and biological parameters may possess potential as early and sensitive indicators for soil ecological stress and soil health assessment (Schloter et al., 2018). Soil micro-organisms play a crucial role in carbon and nutrient cycling in ecosystems. Soil microbial biomass (living part of soil organic matter) is the total microbial composition and is considered an important indicator of the soil quality index of soil fertility, which depends primarily on the rate of nutrient transformation and its availability, as well as the quality and quantity of organic inputs (Bending et al., 2004). It acts both as a source(s) and sink(s) of available nutrients and plays a critical role in nutrient transformation in terrestrial ecosystems (Jacoby et al., 2017). Based on the overall discussion that comes under the wider arena of the topic, a conceptual diagram for highlighting the scopes of soil health as an indicator of ecosystem resilience and stability has been presented (Figure 2).

#### Soil bio-indicators related to nutrient cycling in soil ecosystems

Although it is well-established that soil ecosystem functions are likely to be promoted with increased soil microbial diversity, the linkage between microbial diversity and carbon processing in the soil is often criticized based on the concept of functional redundancy (Maron et al., 2018). To that end, the use of microbial physiological indicators (e.g., carbon use efficiency and microbial turnover rate) are recently being promoted to evaluate the warming response of soil microbial community and subsequent decomposition of soil organic matter, SOM (Frey et al., 2013; Hagerty et al., 2014; Sihi et al., 2018; and Walker et al., 2018). These microbial indicators are increasingly being incorporated into microbial and enzyme-based models for predicting SOM decomposition under warming conditions (Allison et al., 2010; Sihi et al., 2016).

The microbial biomass in the soil is generally represented in terms of microbial biomass carbon (MBC), nitrogen (MBN), and phosphorus (MBP). Microbial biomass carbon (MBC) is a relatively small (1–4% of the total SOC pool), labile fraction that quickly responds to carbon availability and is also strongly influenced by the crop management practices and system perturbations usually followed (Verma et al., 2010). It indicates the soil's ability to store and recycle nutrients and energy, and it also serves as a sensitive indicator of change and future trends in organic matter levels and equilibrium. Microbial biomass nitrogen constitutes a significant part of the potentially mineralizable-N and serves both as the transformation agent and source sink of N (Li J. et al., 2019). Consequently, the MBN may have significant impacts on N availability to plants and overall soil N cycling (Singh et al., 2009). Micro-organisms are an integral part of the soil phosphorus (P) cycle and, as such, play an important role in mediating the availability of P to plants. The microbial biomass phosphorous (MBP) is estimated to be around 2–10% of total soil P; however, during different stages of soil development and within litter layers (soil surface), this may be as high as 50% (Achat et al., 2010). The rapid turnover of P in the microbial pool may contribute a major source to the available P pool, as P released from microbial biomass is highly available for plant uptake, as well as the microbial immobilization of inorganic-P protects the P from physico-chemical fixation (Oberson et al., 2001).

Soil with a relatively higher organic matter usually develops larger microbial biomass. Since microbial biomass affects soil fertility and, hence, ecosystem functioning, the measurement of microbial biomass, activity, and nutrient levels have attracted considerable attention to studying complex biogeochemical soil nutrient cycling for nutrient availability. Apart from this, polysaccharides secreted by microbes help in toil aggregation, thereby playing an important role in improving the soil structure as a binding agent. The role of soil microbial biomass in nutrient flow, organic matter turnover, and soil structural stability has led soil microbiologists to use it as a tool for soil management and perturbation studies (Csitári et al., 2021). Thus, microbial biomass could be considered an important parameter for the assessment of soil functional status and soil health as a whole.

Microbial biomass C and N mineralization capacity have primarily been used to estimate changes in soil health due to management and use, whilst dehydrogenase activity, which is a general measure of viable microorganisms, has also been employed in soils affected by heavy metals and pesticides, as well as for the diagnosis of the degree of recovery of degraded soils. The microbial biomass C increased with the intensity of grazing in meadow soils (Banerjee et al., 2000) and with the cereal-pasture rotation (Verma et al., 2010), decreased when the soil was cultivated (Caldwell et al., 1999), and the effect of zero-tillage was not clear (Dalal, 1998). Furthermore, it increased in soils supplied with organic fertilizers (Dalal, 1998), showed an erratic behavior for inorganic N fertilization (Singh and Singh, 1993; Ladd et al., 1994), and did not react coherently to the presence of herbicides (Voos and Groffman, 1997). Moreover, microbial biomass could not be considered a good indicator of heavy metal toxicity in soils (Dalal, 1998). In laboratory experiments, it has been observed that soil microbial biomass decreases with the addition of Cd or Cu. The effect of heavy metals on microbial biomass is not very clear and depends on the nature and concentration of heavy metals. In general, at low concentrations, the effect may not be very pronounced; however, at high concentrations, the heavy metals reduced the content of microbial biomass (Yuangen et al., 2004).

Other potential bio-indicators like kinetic analysis (V_max_ and K_m_) of nutrient-degrading enzymes (carbon, nitrogen, and phosphorus) can shed light on the stoichiometric controls of SOM decomposition under warming conditions (Sihi et al., 2019). A systemic impact of nutrient limitation on soil microbial functions can be revealed by more advanced multi-omics techniques such as community proteogenomics (Yao et al., 2018). A shift in soil microbial biomass carbon:nitrogen:phosphorus (C: N:P) ratio can also be used to evaluate the effect of nutrient limitation in a system, where nutrient limitation in the soil can influence microbial biomass stoichiometry plasticity (Griffiths et al., 2012; Hartman and Richardson, 2013; Fanin et al., 2017). Other important environmental disturbances like drought events can be captured by investigating the richness of soil fungal community. However, the presence of biocrust-forming mosses is known to mitigate this response in arid areas (Delgado-Baquerizo et al., 2018). Fire events, which are often associated with warming and drought events in arid lands, can modulate activities of enzymes released by soil fungi, especially N-Acetylglutamate synthase (NAG), with a more pronounced effect with increased intensity and/or severity of fire (Boerner and Brinkman, 2003; Turner et al., 2007; Liao et al., 2013). The size and diversity of specific functional microbial groups, such as arbuscular mycorrhizal fungi (AMF) and nitrifying bacteria communities, also have the potential to characterize the effects of management on the sustainability of soil (Helgason et al., 1998; Chang et al., 2001). Environmental stresses in coastal regions can be quantified using a systematic evaluation of microbial community structure and function, especially those related to salt tolerance (Chambers et al., 2016). For example, a recent study identified specific soil bacterial taxa (Gammaproteobacteria and Bacteroidetes), where the prevalence of those taxa positively correlated with soil salinity and served as ideal bio-indicators for soil and ecosystem responses to sea-level rise and associated salt-water intrusions in saline soils (Rath et al., 2019).

#### Soil bio-indicators as influenced by management practices for ecosystem viability/sustainability

In agricultural production systems, enhanced soil health should be accompanied by high productivity without detrimental effects on the environment (Govaerts et al., 2006; Griffiths et al., 2010). Researchers have evaluated the effect of agricultural management systems on soil health. Reduced disturbance systems, such as reduced tillage, generally resulted in improved soil health characteristics. Karlen et al. (1994) reported that surface soil in no-till systems had higher aggregate stability, total carbon, microbial activity, and earthworm populations compared to conventionally tilled soil. Estimated soil loss measured by simulated rainfall collection was two to four times greater in plowed soil compared to no-till. Deciphering the landscape variability along with the soil properties is indispensable to planning land use and a sustainable agricultural system.

It is well-known that there are microbial parameters that have the potential to be used as soil health indicators. The microbial properties (viz., diversity and distribution) are sensitive to agronomic management (Bending et al., 2000; Marx et al., 2001). Therefore, information related to microbial characteristics may be used to study soil health. Microbial characteristics may act as consistent soil health indicators influencing soil quality. Microbial parameters are more likely to be affected by agronomic management practices than biochemical parameters. Bending et al. (2004) highlighted that both biochemical and microbial analyses may be carried out to compare the impacts of management on soil health. Land-use changes have a significant effect on organic carbon, nitrogen, and C: N ratios (Knops and Tilman, 2000), as well as subsequent crop productivity. Some reports suggested that restoration processes had a tremendous impact on the quality of soil (McKinley, 2001). Managing soil ecosystems play a pivotal role in the revival of degraded soils. It is predicted that, if the ecosystem is resilient, there will be no disturbance to the microbial structures, and it will remain as such for some time. To date, many indicators have been considered and tested as potential soil health indicators, and many of these indicators have been successful in a variety of agricultural systems (Glover et al., 2000; Reganold et al., 2001; Bhowmik et al., 2016).

Predominance and succession of soil microbiota are invariably related to temporal and spatial differences, which can be appropriately used as an indicator of soil health. A lot of curiosity exists related to the study of microbial biodiversity and its activity on soil health and quality. Studying microbial diversity with culture-based early techniques had several limitations that can now be removed by using advanced techniques like the use of molecular BIOLOG and techniques, denaturing gradient gel electrophoresis (DGGE), metagenomic community-based approaches, analyzing lipids and phospholipids profile, BIOLOG, etc. Soil microbial activity is different from microbial biomass and diversity. Microbial activity indicates a wide range of physiological activities intimidated by soil micro-organisms that include microbial respiration or soil respiration, metabolic quotient, soil enzyme activities, etc. For example, soil respiration is a function of the total soil biological activity, including microbial activity. Some researchers feel that quantification of soil microbial biomass does not give complete information regarding soil health/quality. It is more useful when used in combination with the quantity of respiration to derive the metabolic quotient, which has been used to provide information on changes to the structure and functioning of soil microbial communities (Wardle and Ghan, 1995).

Many metabolic substances can also be used as soil health indicators such as sterols (Ergosterol), antibiotics, protein (Glomalin or Glomalin-related soil proteins), soil enzymes (phosphatase, urease, sulphatase, and dehydrogenase), etc. Soil enzymes are important attributes that are involved in the dynamics of soil nutrient transformation and make nutrients available to plants. It is proteinaceous and is released by microbes during their growth and activity. Therefore, overall soil microbial activity can be derived from soil enzyme activity (Bhowmik et al., 2019; Kumar et al., 2019) and related to soil health.

The microbial redox system and oxidative activities can be measured through soil dehydrogenase activity using various methods. For example, dehydrogenase activity may increase or decrease with tillage (Bergstrom et al., 1998) depending on the soil management. The application of organic manures, green manures chemical fertilizers, landfill effluents, and industrial waste tends to increase dehydrogenase activity (Bardgett et al., 1996; Langer and Günther, 2001; Dhull et al., 2004). The advantage of using soil dehydrogenase activity as an indicator is that it is not usually affected by heavy metals if present in the sample at lower doses (Yuangen et al., 2004). It has the potential to indicate the extent of recovery of degraded soils. For example, soil contaminated by petroleum spillage can be easily assessed using dehydrogenase activity (Margesin et al., 2000). Likewise, the soil management-induced changes can be assessed through microbial biomass C.

β-glucosidase is the most important enzyme involved in the carbon cycle. So, it is generally used for soil health evaluation. Previous reports (Saviozzi et al., 2001; Mawlong et al., 2021) suggested that β-glucosidase activity decreases significantly in arable soils owing to agronomic management, compared to undisturbed forest or meadow soils. β-glucosidase has a negative relation with agricultural practices (agronomic management). However, some agricultural practices, such as the application of organic manures, may increase the β-glucosidase activity (Mawlong et al., 2021), which may lead to an erroneous interpretation of results. Thus, its value as an indicator of soil quality is reduced. Another soil enzyme, urease, which is responsible for the catalysis of urea (urea hydrolysis), is generally not considered for measurement of soil quality as its activity is highly subjective to soil amendments (Chakrabarti et al., 2000), as well as soil management (Saviozzi et al., 2001; Mawlong et al., 2021).

By considering individual indicators or groups of indicators, soil health can be assessed; however, some researchers also developed indices based on the combination of biological indicators to evaluate soil health. For example, the Biological Index of Fertility (BIF) involves the measurement of respiration and enzymatic activities for its calculation (Vittori Antisari et al., 2021). Microbial index of soil (Mi) involves the measurement of microbial biomass Carbon (C) and Nitrogen (N), potentially mineralizable N, soil respiration, bacterial population, mycorrhizal infection, dehydrogenase, and phosphatase activities, and it is derived by Combining all the data with a crop index and nutrient index (Kang et al., 2005). Similarly, the Enzyme Activity Number (EAN) involves dehydrogenase, catalase, alkaline phosphatase, amylase, and protease enzymes (Beck et al., 1984). Soil Biological Fertility Index was proposed for soil monitoring (Pompili et al., 2008; Renzi et al., 2017), and it is based on soil organic matter (SOM = SOC × 1.724), basal respiration (C_bas_), cumulated respiration (C_cum_), microbial biomass carbon (C_mic_), and metabolic quotient (qCO_2_). This indicator is more precise and sensitive compared to other microbial and enzymatic activity (Pompili et al., 2008; Renzi et al., 2017).

Besides the soil parameters related to microbial activity, soil organic carbon content and its different fractions/ pool can widely be used for soil health measurement alone; however, its response to actual management practices is often slower than biological activity, which is closely linked to microbial activity. Therefore, it was believed that the activity of microorganisms involved in the carbon cycle is a better indicator than the carbon content itself (Smith, 2004; Marinari et al., 2006). In the study of soil carbon, labile carbon pools are widely used for soil quality assessment and will be a better and more sensitive indicator for soil quality. More often, SOC and its lability are highly recommended as an indicator of soil sustainability as it is associated with short-term nutrient cycling (Mtambanengwe and Mapfum, 2008; Bhowmik et al., 2017a). Different methods are used to measure the labile pools of carbon, as well as carbon fractions for the soil quality determination. Labile carbon and its relative proportion to total organic carbon content are also widely used as the index called Carbon management index (CMI), which is determined based on the labile carbon content and its proportion over the total carbon (Blair et al., 1995; Verma et al., 2010). Labile carbon determination varies depending on the method used; its values also vary, however, and it can also be considered as the soil quality indicator for most of the studies.

#### Bio-indicators related to nitrogen cycling in soil ecosystems and aquatic environment

Soil health-promoting management practices enable synergy between multiple soil functions and the prospect of soil-based ecosystem services, in terms of crop production, nutrient recycling, water infiltration and purification, and climate moderation. Worldwide, only half of the nitrogen (N) fertilizers are taken up by crops, thus, a particularly critical soil health need is to improve N use efficiency by understanding the microbial N transformations that help synchronize N availability with plant uptake, and in turn, decrease N losses from soil. Nitrification is the most important component of the biogeochemical N cycle and involves the oxidation of ammonia to nitrite, and then, to nitrate by groups of micro-organisms known as nitrifiers. Nitrification increases the likelihood of N loss from soils. In ammonia oxidation, the first step of nitrification is catalyzed by the *amoA* gene encoding the α-subunit of the ammonia monooxygenase enzyme. Ammonia oxidation is completed in two steps: by two distinct groups of nitrifiers, namely, ammonia-oxidizing bacteria (AOB) and ammonia-oxidizing archaea (AOA). These two groups have shown huge potential as molecular bio-indicators.

Like the agroecosystem, water bodies and the aquatic environment are also interrelated to each other and depend on the activities associated with our agricultural management practices. The use of fertilizers, pesticides, and other agrochemicals has an immense impact on the aquatic environment. To quantify the changes in the aquatic environment, we also need some sensitive indicators to evaluate them. The main consequences of human activities are the release and addition of different organic compounds, pollutants, pesticides, agrochemicals, antibiotics, etc. (Sauve and Desrosiers, 2014; Geissen et al., 2015). Besides the addition of different chemicals, other stresses were also developed in the aquatic environment (like raise in temperature, acidification, acid mine drainage, etc.). In earlier times, different indicators like fish, invertebrates, aquatic animals, and animals are used to monitor the change in the aquatic environment (Li J. et al., 2019). After the presence of particular bacteria, fungi are also used to monitor changes in the aquatic environment. However, these indicators have several limitations like being unable to culture in the laboratory condition, microscopic counting, etc. Besides, in recent days, analysis of the microbiome (microbial community structure, diversity, and patterns) is used to assess the aquatic environment (Michan et al., 2021). In the heavy metal contamination site, the proliferation of resistant bacteria, and bacterial genes was used to quantify the impact (Thomas IV et al., 2020). Microbial abundance, diversity, and activity are significantly affected by the surrounding environment and are highly sensitive to natural and anthropogenic activities, hence, perfect potential indicators of environmental disturbances (Khan et al., 2018; Milan et al., 2018).

However, in recent days, with the development of advanced technologies, DNA-based techniques are being used to detect and quantify the microbes and resistant genes in water samples. Different technology like metagenomic analysis, 16 S RNA, PCR, and qPCR-based analysis are used for evaluation. Microbes have an impact on environmental changes, hence, based on these facts, mass spectrometry imaging (MSI) has been considered a general tool to study micro-organisms (Maloof et al., 2020). PCR-based techniques are also being used to study the molecular viral as they are sensitive, as well as highly specific.

Computational tools and bioinformatics are also employed as environmental monitoring tools, such as pesticide bioremediation. It is executed through an online platform of biodegradative (open access) databases and necessary information provided on biodegradation pathways, as well as microbes-mediated biodegradation of xenobiotic (pesticide) molecules (Nolte et al., 2018). These databases include the University of Minnesota Biocatalysis/Biodegradation Database (UM-BBD), Biodegradation Network-Molecular Biology database (Bionemo), Pesticide Target interaction database (PTID), Microbial Genome Database (MBGD), Biodegradative Oxygenases Database (OxDBase), BioCyc, and MetaCyc for both windows, as well as Linux operating systems (Arora and Bae, 2014). Another new bioinformatics approach, BarcodingGO, was developed by a team of scientists from Brazil, aiming for environmental DNA and bioinformatics as an environmental monitoring tool under simulated conditions (Nunes et al., 2021). This was performed through a biodiversity survey (analyzing DNA released by organisms living in a specific environment) to measure the impact of an environmental disaster, identified by the unique quick response (QR) codes that represented pre- and post-scenarios of environmental disaster.

A comprehensive and easy-to-read information table, encompassing different soil types and their bio-indicators, is presented (Table 1).

**Table 1:** Important bio-indicators identified for different soil ecosystems.

**SI. No**.	**Soil or management practices**	**Identified bioindicators**	**References**
1.	Agricultural management practices or environmental changes	Microbial biomass C, functionality and diversity of microbial communities, metabolic quotient (qCO_2_), microbial quotient (qMIC), enzyme activities, mineralizable carbon, nitrogen, phosphorus and sulfur, soil respiration, soil organic matter/carbon and labile carbon pools, Carbon management index (CMI), Biological Index of Fertility, Microbial index	Blair et al. (1995); Andersen and Sparling (1997); Kang et al. (2005); Pompili et al. (2008); Verma et al. (2010); Gómez-Sagasti et al. (2012); Bhaduri et al. (2017a,b); Renzi et al. (2017); Bhowmik et al. (2019); Kumar et al. (2019); Li L. et al. (2019); Vittori Antisari et al. (2021)
2.	Land use intensification, different fertilizer history, Pollution of agrochemicals	Soil microbial community (composition and structure), soil enzyme activity, 16S rRNA amplicon sequencing based operational taxonomic units (OTUs)	Gomez et al. (2004); Saha et al. (2015, 2016a,b); Gaind and Singh (2016); Pérez-Brandán et al. (2016); Sahoo et al. (2016); Mahapatra et al. (2017); Sihi et al. (2017); Xu et al. (2017); Kumar et al. (2018)
3.	Exposure of soils to trace (or, heavy) metals, organic pollutants, mine tailing, radioactive waste	Earthworm, acidophilic bacteria, stress proteins (hsp70 and hsp60), functional genes related to fatty acid metabolism (*acc, fab*, and *fad* genes), Shannon diversity index, metabolic quotient and cellulase activity, urease and invertase enzyme activities, nematode diversity, maturity index, cryptogamic biota (lichens and bryophytes)	Coleman et al. (1998); Korthals et al. (1998); El-Bestawy et al. (2005); Ciarkowska et al. (2014); Galitskaya et al. (2015); Patel et al. (2016); Huggett (2018); Rola and Osyczka (2018)
4.	Gold mining in tropical rainforests	Ratio of soil denitrifying enzyme activity and substrate induced respiration	Schimann et al. (2012)
5.	Radioactive pollution, contaminated with radionuclides	Earthworms, millipedes, collembolans and oribatid mites, land snails, aquatic mosses	Gaso et al. (1995); Hongve et al. (2002); Zaitsev et al. (2014); Rybak et al. (2021)
6.	Wetland pollution and restoration	Microbial diversity and community comparison, Ratios of oligotrophic:copiotrophic organisms such as the ratio of ammonia-oxidizing archaea (AOA) to ammonia-oxidizing bacteria (AOB)	Dziock et al. (2006); Martens-Habbena et al. (2009)
7.	Warming response of soil and effect of climate change	Microbial physiological indicators (carbon use efficiency, microbial turnover rate), kinetic analysis (V_max_ and K_m_) of nutrient (carbon, nitrogen and phosphorus) degrading enzymes	Frey et al. (2013); Hagerty et al. (2014); Sihi et al. (2018, 2019); Walker et al. (2018)
8.	Sandy ecosystems	Soil nematode communities	Guan et al. (2015)
9.	Fire	N-Acetylglutamate synthase (NAG)	Boerner and Brinkman (2003); Turner et al. (2007); Liao et al. (2013)
10.	Sea level rise and salt-water intrusions	Soil bacterial taxa (Gammaproteobacteria and Bacteroidetes)	Rath et al. (2019)
11.	Aquatic environment	Fish, invertebrates, aquatic animals, microbiome (microbial community structure, diversity and patterns), metagenomic analysis,16s RNA, PCR and qPCR-based analysis, mass spectrometry imaging	Li J. et al. (2019); Maloof et al. (2020); Michan et al. (2021)

### Role of ammonia oxidizers as soil bio-indicators sensitive to soil management practices and environmental conditions

Assessments of the ammonia oxidizer community have revealed that the AOA and AOB not only respond differently to abiotic and biotic soil characteristics (niche specialization) but also possess different patterns of nutrient utilization (niche differentiation) (Zhalnina et al., 2012). Metagenomic studies have enabled us to realize that these two assemblages have different substrate (ammonia) affinities like ammonia, AOA has higher affinity and substrate toleration (Prosser and Nicol, 2012). Acidic soil pH has been reported to support more AOA as compared to AOB communities (Nicol et al., 2008); however, results might vary with geographical location and site differences (Jiang et al., 2014). Metabolically, ammonia oxidizers have always been considered to be mainly autotrophs (Hallam et al., 2006). Recent evidence from agricultural soils supports the fact that the potential to assimilate organic compounds (i.e., heterotrophic or mixotrophic metabolism) can be more prevalent in AOA than in AOB (Xue et al., 2016).

Quantitative PCR (qPCR) analysis and nitrification kinetic studies are used to measure AOA and AOB, and their relative contribution to nitrification in agricultural soils. The relative contribution of AOA and AOB communities responding to nitrification might differ in agricultural soils. AOA co-exists with AOB in agricultural soil but responds differently to climatic conditions and nitrogen management (Kowalchuk et al., 2000; Taylor et al., 2012, 2013; Habteselassie et al., 2013; Jiang et al., 2014; Banning et al., 2015; Giguere et al., 2015; Bhowmik et al., 2016; Ouyang et al., 2016). There has been evidence in the literature that AOB communities, as compared to AOA, are more responsive to the application of N fertilizer and have dominated nitrification in most agricultural soils except for acidic soils (Jia and Conrad, 2009; Di et al., 2010; Zhang et al., 2010; Xia et al., 2011; Ai et al., 2013; Giguere et al., 2015; Ouyang et al., 2016; Song et al., 2016). On contrary, a recent study by Orellana et al. (2018) on soil metagenomes from agricultural soil showed the increased response of AOA populations to synthetic N fertilization as compared to AOB. All these above-mentioned studies have vastly benefitted directly or indirectly from the sequence database generated with next-generation sequencing (NGS).

The literature is replete with studies that suggest the potential of the nitrifier and denitrifier gene copy numbers to be sensitive indicators of key management practices and are influenced by the amount of available nitrogen (N) and carbon (C), and climatic factors, such as soil temperature and moisture. Long-term organic management systems (20 years) increased the microbial diversity resulting in enhanced N mineralization potential (Berthrong et al., 2013). In acidic soil in China, the community composition of AOA, and not AOB, was affected due to fertilization (He et al., 2007). This trend was reversed in alkaline soil under similar fertilization regimes (Shen et al., 2008). In a 44-year-old grassland fertilizer experiment, organic input (cattle slurry) increased *amoA* gene of ammonia-oxidizing archaea (AOA) significantly, whereas chemical N fertilization increased the ammonia-oxidizing bacteria (AOB) population (Zhou et al., 2015). Rudisill et al. (2016) also reported that organic fertility management practices stimulate nitrification, which is mainly reflected by increased AOB activity. In Zn contaminated soil with the recovery of nitrification activity, a shift in the community structure of AOB was observed without any observable difference in AOA (Mertens et al., 2009). Previous studies by Wessén et al. (2011) and Tsiknia et al. (2015) across a diverse range of management practices and climatic conditions suggested that *amoA* gene from functional microbial communities of AOA and AOB was an effective biological indicator. In biogeochemical cycling, however, some studies suggest that the direct effect of management regulates nutrient availability and not necessarily soil microbial activity or diversity (Wood et al., 2015).

Bio-indicators, e.g., nitrification potential, as well as *amoA* gene, copy numbers of AOA and AOB, also respond to interactions between soil abiotic and biotic properties (Fortuna et al., 2012). Several meta-analyses have concluded that both AOA and AOB populations responded to the application of N (Carey et al., 2016; Ouyang et al., 2018). Robinson et al. (2014) showed that soil pH regulated AOA and AOB in urine-treated soils. A study by Höfferle et al. (2010) showed that sewage-polluted soil increased the abundance of AOB over AOA. Mundepi et al. (2019) reported that in poultry-litter-amended soils, an abundance of AOB decreased, whereas AOA increased. However, functionally, AOB was contributing more to nitrification despite lower populations as compared to AOA. Response of AOA and AOB to N addition and soil moisture conditions in a typical temperate steppe suggested that AOA and AOB had distinct ecological niches, and AOB was more sensitive to N and precipitation and was the main driver of nitrification in such soils (Chen et al., 2013). A study was conducted by Ouyang et al. (2016) to measure the response of ammonia-oxidizing nitrifiers in agricultural soils treated with ammonium sulfate or steer waste compost. The AOB, as compared to AOA, contributed to nitrification potential in these N fertilized soils. A study by Giguere et al. (2015) revealed that changes in AOB populations were more sensitive to net nitrification rates in cropped soil, whereas AOA populations responded more to non-cropped soil. Banning et al. (2015) reported that in semi-arid agricultural soil, *amoA* AOB dominated nitrification activity and was four-fold higher than *amoA* AOA gene copies in the 0–10-cm soil layer. In a 2-year field experiment, Muema et al. (2016) observed that the AOB was more sensitive to organic inputs, whereas AOA to chemical fertilization. On the other hand, Shen et al. (2015) reported that the long-term application of organic manures with or without mineral NPK fertilizer increases the population of AOA in acidic red soil. However, other studies observed that long-term or short-term manure fertilization increased the populations of AOB rather than AOA in paddy soil (Wang et al., 2014). Ammonia oxidizers are also sensitive to substrate concentration in the soil. High ammonia substrate concentration favored AOB, whereas low ammonia substrate conditions favored AOA in grassland soils treated with animal waste (Di et al., 2010). Liu et al. (2016) found that ammonia-oxidizing populations of AOA and AOB also responded to the difference in the intensities of grazing in a semi-arid grassland. However, another study found that in N-limited natural grasslands, AOA is an important driver of nitrification, whereas in N-amended grassland soils, the contribution of AOB toward gross nitrification is more dominant (Sterngren et al., 2015). Radl et al. (2014) reported that management practices like cattle overwintering could also be a major factor that could alter the AOA vs. AOB populations. Their study concluded that the AOA populations dominated in no grazing, whereas the AOB outnumbered AOA in severely grazed sites.

Ammonia oxidizers are also effective indicators of the amount of oxygen present in the soil. Liu et al. (2015) observed that AOA rather than AOB had better adaptability in flooded soils where the oxygen concentration was lower. Another group of researchers also reported that AOB was more sensitive to oxygen availability as compared to AOA (Ke et al., 2015). Under simulated stress conditions like drought (drying-wetting cycle) in grassland soil that rarely experience drought, both the AOA and AOB populations showed poor resistance and resilience (Thion and Prosser, 2014). However, a study conducted by Yarwood et al. (2013) reported that AOA had the potential to persist in a 12-year soil ecosystem with no organic matter input. To determine the resilience of nitrification activity under increased acidity levels, Sher et al. (2013) found that in arid and semi-arid soils under increased acidity levels, AOA was predominant during water stress conditions and in higher temperatures, whereas the AOB was predominant in humid conditions with higher precipitation.

### Impact of bio-indicators on crop productivity: A global scenario

Intensive agricultural management has contributed to economic and social development. However, it is also responsible for land degradation, biodiversity loss and ground water depletion, and contamination (Kirschenmann, 2010). The United Nations Environment Programme (UNEP) sponsored project, the Global Assessment of Soil Degradation (GLASOD), estimated that agricultural land has undergone soil degradation to an extent of 38%. In a certain type of soil, it has been observed that crop productivity has declined 3–12 times in degraded soils compared to normal soils (Kyawt et al., 2015). Among the crops, rice and wheat show the steepest decline in crop productivity in degraded/erosion-prone soils (Panagos et al., 2018). Without major capital investments and engineering inputs, agriculture has become unsustainable. More recently, there have been concerns about the maintenance of crop productivity and environmental quality to maintain the human population in the future (Liu et al., 2006). Tools for assessing soil health are required to ensure sustainable agriculture for future generations by evaluating the effects of management practices on soil processes. Soil quality or soil health has been considered an indicator of crop productivity (Doran and Parkin, 1994; Acton and Gregorich, 1995; Karlen et al., 1997). Crop productivity has been traditionally linked to soil health, wherein it is assumed that improvements in soil health will alleviate growth-limiting factors and, hence, improve yields (Miner et al., 2012).

Evaluation of soil health should involve physical, chemical, and biological soil properties (Bhardwaj et al., 2011). Until now, changes in physical, chemical, or biological soil properties are monitored for assessing soil quality. Many researchers believe that combining several indicators into a single index would give a better perception of soil health rather than individual parameters (Sharma et al., 2005). Some others feel that only the linked parameters are important soil functions that should be combined for the calculation of indices to predict the sustainability or productivity of an agro-ecosystem (Herrick, 2000). It is difficult to develop a single universally accepted standard for each indicator as the soils, as well as management practices, vary widely. It is even more difficult to combine these indicators to derive an index that is applicable to all soil types or agroecosystems. Despite the emphasis on multi-parameter indexing (Doran and Parkin, 1994; Diack and Stott, 2001; Mandal et al., 2008; Sharma et al., 2008; Bhardwaj et al., 2011), many researchers like Visser and Parkinson (1992) believe that laboratory procedures for determination of decomposition rates, microbial biomass C, and soil enzymes are efficient and can be used independently for assessment of soil health.

To date, many quantifiable soil quality indices have been developed by many researchers. Larson et al. (1994) established the concept of a Minimum Data Set (MDS). They recommended the importance of identifying the set of indicators that are more sensitive to a particular soil management practice and only the selected indicators should be considered and used to assess the sustainability of the management practice in question. Doran and Parkin (1994) worked on the same line and expanded the minimum data set of Arshad and Coen (1992) by including biological properties that were not included earlier. However, DuPont et al. (2021) suggested that soil health indicator minimum data sets should be regional and management of goals that specifically identify and quantify the factors that affect crop productivity.

Soil biological properties are sensitive, essential, and give more variable results. Therefore, biological properties are indispensable when considering the characterizing of soil health (DuPont et al., 2021). Among the bio indicators, only microbial biomass C is being used as the only biological parameter for calculating the soil quality index. The terrestrial SOC is a major pool of soil organic matter. Emphasized that SOC plays a major role in determining the degree of soil erosion. Soil microbial respiration and nutrient cycle are directly dependent on SOC. Other soil health indicators directly dependent on SOC are available to water (Hudson, 1994), infiltration rate and capacity (MacRae and Mehuys, 1985; Pikul and Zuzel, 1994), and soil aggregate formation and stability (Oades, 1984; MacRae and Mehuys, 1985; Tisdall and Oades, 2012). Soil organic carbon or SOM is directly linked to many other soil health indicators, as well as crop productivity (National Research Council, 1993; Cannell and Hawes, 1994; Larson et al., 1994). SOM is one of the few indicators that relate to both soil health and crop productivity (de Lima et al., 2008; Van Eekeren et al., 2010; Hanse et al., 2011). Doran and Parkin (1994) emphasized the role of cropping systems, along with soil management systems and their impact on SOM. It is reported that even if SOM is not correlated to crop productivity goal, it must be included in the minimum data set as it influences multiple soil functions, such as microbial activity, nutrient cycling, soil carbon accumulation, and water relations (DuPont et al., 2021).

The invertebrates have also been used as indicators of soil health but, to date, no universally accepted criteria for its use have been found. In the same line, Linden et al. (1994) worked on earthworms, which are the most widely used invertebrate as soil indicators. They reported that the determination of earthworm activity in some soils may be relevant but not crucial. It was suggested that earthworms play vital roles in processes like water infiltration and crop root aeration and development, but earthworms are not obligatory for these processes, and high-quality soils may exist even in absence of earthworms.

### An account of “resistance and resilience” of bio-indicators

Resistance is the property of an ecosystem to remain “essentially unchanged” when subjected to disturbance/stress. However, resilience refers to the capacity to recover from disturbance or withstand ongoing pressures/stress. It is a measure of how well an ecosystem can tolerate disturbance(s) without collapsing. Resistance can be measured just after the disturbance, whereas resilience can be assessed progressively with time after the disturbance, i.e., can only be determined after the disturbance has ceased. A major goal in restoration is to restore both resistance and resilience so that the restored entity is self-perpetuating and does not require ongoing interventions to be sustainable (Lake, 2013). Ecological restoration is widely practiced for rehabilitating ecosystems and habitats that have been degraded or impaired through human use or other causes. In other terms, resilience can also be defined as the capacity of a system to absorb disturbance and re-organize in ways that retain essentially the same functions, structures, identities, and feedback (Walker et al., 2004). This includes two important mechanisms of resilience, namely, resistance to change and recovery from change.

Studying soil resistance and resilience is another new and emerging facet of assessing soil health. In a study conducted by Griffiths et al. (2005), both physical and biological stability and resilience were studied for soils treated with Cd, Cu, and Zn, digested or undigested sewage sludge. They found that the rate of mineralization of DOC released by alternate drying/wetting cycle was reduced by Zn contamination, while biological resilience was increased in the Zn-contaminated soil and reduced by Cd contamination. The effects of metals (Cd and Cu-contaminated) on physical resilience, in terms of expansion indices (indicating soil aggregation), were greater than the effects on soil C. In another study after imposing heat stress at 42°C for 24 h, the abundance, structure, and activity of two specialized soil bacterial functional groups (denitrifiers and nitrite oxidizers) were studied periodically to assess resistance and resilience (Wertz et al., 2007). The behavior showed differential results while the nitrite oxidizers were more affected, but reducing the diversity of both groups did not impair either their resistance or their resilience following the disturbance. Kumar et al. (2014), in another study, found that the combined application of NPK (balanced) and FYM (from a long-term experimental soil under maize crop) was most effective in enhancing resistance and resilience of soil microbial activity in terms of substrate-induced respiration and dehydrogenase activity against heat stress imposed at 48°C for 24 h. Here, an attempt was made to accumulate the important information generated from soil resilience models for uplifting the overall crop productivity and environmental quality (Table 2).

**Table 2:** Soil resilience model for improving agricultural productivity and environmental quality.

**Study site**	**Targeted stress environment**	**Crop/cropping system**	**Implications**	**References**
1. N-E Nile Delta, Egypt	Soil degradation by waterlogging salinization, and alkalinization	Irrigated and rainfed croplands	Soil degradation processes dominated over the soil resilience causing a decline soil productivity index by 45.82% of the total area over a span of 35 years	Kawy and Ali (2012)
2. South-central region of the State of Parana', Brazil	Long-term tillage impacts vs. continuous no-till	Soybean-maize system	Increasing labile C fractions under continuous no-till has been reflected for highest resilience index and productivity	de Moraes Sa et al. (2014)
3. Gongzhuling, Jilin province, China	Long-Term fertilization trials	Maize	Organic matter (FYM and straw) amendments mitigated the climate change effects on crop production by enhancing soil resilience and showing better SOC, nutrients and soil water storage	Song et al. (2015)
4. Southern England, Rothamsted research station and nearby areas	Physical stress (uniaxial compaction) and biological stress (transient heat or persistent Cu stress)	Arable and grasslands	OM and clay content critically determined the soil resilience; grassland soils were more resilient to both physical and biological stresses than the arable soils	Gregory et al. (2009)
5. IARI, New Delhi, India	Short-term heat stress imposed at soils of long-term fertilization trials	Maize	NPK + FYM was most resilient against heat stress in terms of soil microbial activity (substrate-induced respiration and dehydrogenase activity)	Kumar et al. (2014)
6. Scottish Agricultural College, Auchincruive Estate, Scotland	Heavy metal (Cd, Cu, Zn)-contaminated sewage sludge	–	Medium-Term (9 years after application) effects of metal-contaminated sludges on the stability and resilience of a sandy clay loam soil. The most obvious effect of sludge addition was an increase in soil C content, but there were no significant effects of metals on soil C. Medium-Term (9 years after application) effects of metal-contaminated sludges on the stability andresilience of a sandy clay loam soil. The most obvious effect ofsludge addition was an increase in soil C content, but therewere no significant effects of metals on soil C 9 years of metal-contaminated sludges reflected on the resilience, increase in soil C content was prominent by applying sludge while metal contamination disturbed the C- metabolism and physical resilience	Griffiths et al. (2005)
7. Inner Mongolian Grassland Ecosystem Research Station, Chinese Academy of Sciences	Mechanical (physical) stresses imposed under arable systems, including vehicle traffic	Ungrazed and undisturbed soils	Mechanical resilience of fine-textured sandy loam soil was improved by adding woodchip biochar, with further impact on stability, compressive behavior and cohesion. Better proportion of medium to fine pores and improved water retention was noticed	Ajayi and Horn (2017)
8. Rubite, Granada, Spain	Organic waste (olive-mill solid waste) and its vermicompost was applied to a degraded soil	Marginal agricultural lands	Soil resilience factors of the disturbed soil like the amplitude (*period of recovery*) and the elasticity *(speed of recover*y) to the initial state after disturbance, were successfully monitored by o-diphenol oxidase, β-glucosidase and dehydrogenase activities. Vermicomposting promoted microbial activities in the degraded soil without any toxicity effects	Benitez et al. (2004)

### Ecosystem restoration *vis-à-vis* sustainable development goal

The 2030 Agenda for Sustainable Development, adopted by all United Nations Member States, prepared a blueprint in 2015 for peace and prosperity for all people and the planet earth, for present and future scenarios. There are a total of 17 Sustainable Development Goals (SDGs), which are recognized for ending poverty and other deprivations with strategies that improve health and education, reduce inequality, and spur economic growth– simultaneously while tackling climate change and working to preserve our oceans and forests. Addressing the SDG has been an urgent call for action by all countries, both developed and developing, and in a global partnership.

It has been noticed and reported by several researchers around the world that key ecosystem renders numerous essential services to food and agriculture, including supply of freshwater, protection against hazards, and provision of habitat for species, such as fish and pollinators, which are diminishing rapidly. Moreover, the degradation of land and marine ecosystems has taken a toll on the wellbeing of 3.2 billion people and charges about 10 percent of the annual global GDP due to the loss of species and ecosystem services. Among the 17 goals, the SDG 15 of the 2030 Agenda for Sustainable Development is devoted to “*protect, restore and promote sustainable use of terrestrial ecosystems, sustainably manage forests, combat desertification, and halt and reverse land degradation and halt biodiversity loss*” (source: United Nations, 2022).

Considering the importance, UN General Assembly declared “The UN Decade on Ecosystem Restoration 2021–2030,” which aims to upscale the restoration strategies for degraded and destroyed ecosystems as an established measure to combat climate change and enhance food security, water supply, and biodiversity. Further, the successful restoration process of 350 million hectares of degraded land by 2030 could be able to generate 9 trillion US$ in ecosystem services and take an additional 13–26 GT of GHG out of the atmosphere (source: UNEP, 2019).

Ecosystem restoration is considered one of the fundamentals to achieving the SDGs, where several other globally challenging factors like climate change, poverty eradication, food security, water, and biodiversity conservation are closely linked.

The UN Environment, as well as the Food and Agriculture Organization (FAO) of the United Nations, has taken responsibility for the implementation of the UN Decade on Ecosystem Restoration and has prioritized the following areas in this regard (source: UNEP, 2019):

i. Innovations on biodiversity and land degradation
ii. Protection of the marine environment from land-based activities
iii. Protecting the ecological balance of food chains by conserving and sustainably using mangrove ecosystems
iv. Sustainable coral reefs management
v. Deforestation and agricultural commodity supply chains
vi. Sustainable nitrogen management
vii. Rangelands and pastoralism
viii. Sustainable blue economy
ix. Sustainable peatland management for tackling climate change

## Conclusion and way forward

The ecosystem of the earth can be disturbed naturally over time or through anthropogenic intervention. In the changing era of climatic aberrations, chances of land degradation and loss of biodiversity (both above-ground and underground) are unprecedented. Hence, it is crucial to develop a sound understanding of how the recovery of soil ecosystems is possible. The existing and upcoming concepts of function-based soil quality, resistance, and resilience are interlinked processes that may help in ecosystem restoration. Sustainable soil processes as regulated by effective soil indicators can influence both crop productivity and environmental protection. Of late, environmental issues after anthropogenic activities like mining and agrochemical pollution have created much concern and can be monitored from time-to-time using soil bio-indicators. In this review, the latest techniques for measuring function-based soil bio-indicators for both arable soil and aquatic wetlands were discussed in detail, which shows the possibilities for future research and policy intervention. Apart from being the medium of the food production system, the soil is rather considered a crucial substance for protecting the valuable environment, hence, its sustenance is immensely important. Thus, an understanding of resistance and resilience after short- and long-term soil manipulation is equally relevant. Here, three interconnected aspects of soil quality, soil sustainability, and ecosystem restoration were discussed, fulfilling the aim of sustainable development goals. Climate change in different forms and degrees has been a real challenge over the years to save the soil and the precious ecosystems surrounding it. So, the chain of soil's ecosystem restoration capabilities is an important global issue where the functionality, behavior, and achieving sustainability goals are immensely important to remark and apply for maintaining a healthy soil for the generations to come.

## Author contributions

DB conceptualized the idea. DB and DS outlined the review. DB, DS, and AB took the lead in drafting the manuscript with the help of other co-authors. BV, SM, and BD supplemented in manuscript preparation. AB, BD, and BV did further editing and technical checking in the manuscript. DB and BD produced visualization. All authors contributed to literature collection and compilation of information, and contributed to the article and approved the submitted version.

## Funding

This work was partially supported by the United States Department of Agriculture-National Institute of Food and Agriculture (USDA-NIFA Evans Allen NC.X332-5-21-130-1 accession no. 1023321 and USDA-NIFA Grant No. 2019-51106-30188–SUB00002201).

## Conflict of interest

The authors declare that the review was drafted in the absence of any commercial or financial relationships that could be construed as a potential conflict of interest.

## Publisher's note

All claims expressed in this article are solely those of the authors and do not necessarily represent those of their affiliated organizations, or those of the publisher, the editors and the reviewers. Any product that may be evaluated in this article, or claim that may be made by its manufacturer, is not guaranteed or endorsed by the publisher.
